# PD198 A Review Of Regulatory Theory To Inform Discussion On Aligning Regulation And Health Technology Assessment Of Medical Devices

**DOI:** 10.1017/S0266462324004203

**Published:** 2025-01-07

**Authors:** Kathleen Harkin, Jan Sorensen, Steve Thomas

## Abstract

**Introduction:**

If regulation and health technology assessment (HTA) are to be aligned, we must understand regulation and regulatory policy goals. Furthermore, regulatory policy is most likely to achieve its goals if it is informed by theory and underpinned by evidence. However, regulatory theories are rarely explored by HTA bodies. Therefore, this study explored regulatory theories as they relate to medical device regulation.

**Methods:**

Literature describing regulatory theories was reviewed and synthesized narratively to explain what is meant by regulation and why governments regulate. Competing theories were discussed, reflecting on their relevance to the regulation of medical devices.

**Results:**

The theory of perfect competition suggests that, when key assumptions hold true, market forces will result in the most efficient allocation of resources. However, when they don’t, market failure results. Governments frequently regulate such markets. Stigler suggests that they do so to serve either public or private interests. Market failure is the norm in health care, so it requires regulation. If medical device regulation is intended to serve the public interest, it should be patient-centered, balance market power amongst many producers, reduce information asymmetry, and ensure that only safe and effective devices enter the market. HTA assumes a public interest perspective.

**Conclusions:**

Regulations that maintain the confidentiality of clinical evidence underpinning medical devices and allow them to enter healthcare markets with insufficient evidence of efficacy do not serve the public interest. This raises the questions of whose interests does medical device regulation serve, and can HTA align with this?

